# Book Review

**Published:** 1907-09

**Authors:** 


**Encyclopedia and Dictionary of Medicine and Surgery.** Vols. II., III., IV. Edinburgh: William Green and Sons. 1907.—The succeeding volumes of this great work are following each other without delay. The first volume was noticed in our issue of December, 1906, and thr e others are now before us, carrying the subject matter to the word Intussusception, and completing more than one-third of the whole work. The four volumes now issued contain almost all of the articles of the first five volumes of the *Encyclopædia Medica*, together with a great mass of new material, consisting for the most part of shorter contributions. The names of the authors are a sufficient indication of the quality of their writings, it would be invidious to select any for especial commendation, and the editorial part of the work is beyond all praise. The numerous cross-references have been prepared with great care, and add much to the value of the work as facilitating immediate reference to all the aspects of any given subject.

**A Dictionary of Medical Diagnosis.** By HARRY LAWRENCE McKISACK, M.D. Pp. xi., 583. London: Baillière, Tindall & Cox. 1907.—This work is described by its author as a treatise on the signs and symptoms observed in diseased conditions, for the use of medical practitioners and students. It is therefore an exposition of the language of signs as presented in the bodies of our patients. The author endeavours to avoid the discussion of diseases as they are described in the text-books of medicine, and restricts himself to a description of the various signs and symptoms of disease. The dictionary form is convenient for easy reference, but some of the articles are of considerable length, notably that on blood examination, which gives all needful details with regard to the study of stained films, and the determination of the opsonic power of the serum. The author may well expect to be the teacher over a much wider field than the Royal Victoria Hospital of Belfast.

**On Treatment.** By Harry Campbell, M.B., B.S. Pp. viii., 421. London: Baillière, Tindall & Cox. 1907.—This is not a text-book, but a series of essays, giving the personal point of view of the author on certain questions of therapeutics. The subjects are very varied, ranging from the education and personality of the physician and his proceedure in consultation through a great variety of interesting topics up to the latest fads in diet and therapeutics. From the previous works of the author we should expect a good literary product founded on common sense. We are not disappointed, for the book is full of sound teaching, and is so interesting, that it may well take precedence over the popular novel of the day.

**Gout.** By Arthur P. Luff, M.D. London: Cassell and Co. 1907.—The issue of a third edition of this well-known book shows that gout cannot yet be considered as an extinct disease. In fact, as a source of polemical discussion, it appears likely to afford some interest for another century or two. The enthusiasm for golf and outdoor exercise at one time bid fair to diminish the transmission of this favourite family heirloom, but with the appearance of the motor hopes such as these speedily vanished. Vibration, deficient exercise, increasing appetite, and perpetual faucial dryness all prognose the recurrence of swathed limbs and restrained expressions. Hence it is well to have at hand a summary of recent work. Dr. Luff has considerably enlarged his original monograph, and readers will find a clearly-stated résumé of the present views upon this most difficult subject. It must be admitted that there is still much to be done before the inner metabolic changes in gout are delineated, but it is satisfactory to note that a toxic theory is substituted for the discarded renal theory. Treatment is considered in detail, and a new feature consists of the differential diagnosis in chronic diseases of the joints. There is a very useful table upon the choice of a spa. Bath is recommended as exceedingly good for the absorption of gouty deposits from the joints and tissues. Harrogate is useful for the same purposes, and also for gouty dyspepsia, hepatic symptoms, glycosuria of gouty origin, and gouty skin affections.

**Selected Essays on Syphilis and Small-pox.** Translations and Reprints from Various Sources. Edited by Alfred E. Russell, M.D. Lond. Pp. xii., 215. London: The New Sydenham Society. 1906.—This volume presents several papers of great interest illustrating the progress of current experimental research. It will be found very convenient for reference to have Schaudinn and Metchnikoff's experimental investigations on syphilis, and the studies of Calkin's and Councilman on variola, all work of extreme importance, gathered together into a well-printed and handy volume.

**The Book of Prescriptions** (BEASLEY). Rewritten by E. W. LUCAS. Eighth Edition. Pp. ix., 366. London: J. & A. Churchill. 1905.—The popularity of this book has called for an eighth edition, and there is now an introduction of two pages in length by Dr. Arthur Latham. Dr. Latham says that the book has been written to assist the senior student in his work at the hospital; if so, the senior student must be careful to distinguish pharmacopœial from other preparations, for there is little or no distinction made for him. Should the senior student look up podophyllin for a prescription to assist him in prescribing that drug in a mixture, he will be disappointed, for the four prescriptions given are all pills, and there is no mention of the ammoniated tincture, nor of a suitable menstruum should the pharmacopœial tincture be used. He will be wise if he refrains from calling sodium tartrate sodii citras (p. 279). He should also refrain from learning certain prescriptions of the blunderbuss type, at any rate before he is qualified, for he will find that a prescription containing over twenty drugs will not be thought highly of by his examiners, even though he plead that it is the "Anti-Cholera Mixture, R.C.P." But he may find, when he is qualified, that it is a useful and up-to-date little book, which will often help him to make a suitable mixture, or remind him of a line of treatment which he had forgotten.

**A Handbook of Medical Jurisprudence and Toxicology.** By WILLIAM A. BREND, M.A., M.B., B.Sc. Pp. xiii., 287. London: Charles Griffin & Company, Limited. 1906.—Though this is a small book on a large subject, it is remarkably complete, and is specially interesting from the large number of recent legal cases which it embodies. We have tested it in numerous places, and find that it is on the whole singularly free from omissions, though, from its compressed style, students would not always see the importance of the facts stated. Still, in the space which the author has allowed himself, he writes clearly and with vigour, so that his pages are by no means unattractive, even apart from the cases he cites or describes from his own experience. We shall hope to see an expanded edition later on, which should be an invaluable book. The various chapters are of unequal value. In the treatment of opium-poisoning there is no clear statement of the curious excretion into the stomach of opium or morphine circulating in the blood, which can be removed by repeated lavage with permanganate after an interval. Even morphine injected hypodermically can be thus recovered. Similarly in the treatment of poisoning by arsenic, the quantities of sodium carbonate and perchloride of iron to be used for forming ferric hydrate might have been given in detail. The tests for phosphorus are restricted to Mitscherlich's method, and the dangers of phosphuretted hydrogen are omitted altogether. We should also like to have seen some reference to the much debated precautions ordered by the Home Office as to the glazing of pottery, and finally confirmed under the arbitration of Lord James of Hereford. In fact, the account of several of the important poisons is far too sketchy and brief. A much better chapter, and one of general interest, is the one on " the obligations statutory and moral of the medical man." The law on malpraxis, professional secrecy, undue influence, and death certificates is here explained better than in most treatises of the kind. Indeed, we know of none which sets forth so many recent decisions of importance to the practitioner. Hutchison's useful list of the ingredients of the common patent medicines, such as Cockle's pills and pink pills, is given in an appendix. The book is well printed in clear small type on good thin paper.

**Medical Diagnosis.** By J. J. GRAHAM BROWN, M.D., and W. T. RITCHIE, M.D. Fifth Edition. Pp. xvi., 508. Edinburgh: Wm. Green and Sons. 1906.—The success that this book has obtained is well deserved, and the appearance of the fifth edition is sufficient evidence of its popularity. It is hardly necessary to say more than that this edition has been brought thoroughly up to date, and may be relied upon as a trustworthy and complete handbook for ordinary clinical work. It is profusely illustrated, and the illustrations are good. The additions made add greatly to its value. To those who know the book no further commendation is necessary, but we may add that its convenient size is not the least of its recommendations.

**Elements of Practical Medicine.** By ALFRED H. CARTER, M.D. Ninth Edition. Pp. xvi., 614. London: H. K. Lewis. 1906.— A ninth edition is its own commendation: the book has fully shown its adaptation to the needs of the student of medicine. It has been carefully revised, and has not grown much larger. The very full therapeutic index appears to be needful in these days, when the tendency is to accept the ready-made combinations of the wholesale druggist rather than to devise whatever may be appropriate to the case and the occasion.

**The Etiology and Diagnosis of Epidemic Cerebro-Spinal Meningitis.** By ARCHIBALD WILLIAM TAVES, M.D. Pp. 42. Providence, R.I.: Snow & Farnham. 1906.—This essay won the prize awarded by the Trustees of the Fiske Fund at the annual meeting of the Rhode Island Medical Society in 1906. Its motto, " Keep Watch," is a very appropriate one, inasmuch as epidemics of this disease and sporadic cases also crop up unexpectedly here and there, and diagnosis is difficult until a bacteriological examination of the fluid obtained by lumbar puncture has been made.

**The British Journal of Tuberculosis.** Edited by T. N. Kelynack, M.D. Vol. I., No. 1. January, 1907. London: Baillière, Tindall and Cox.—Another journal has been established in furtherance of the crusade against tuberculosis. This, its first number, contains a series of papers by the best-known writers and authorities. We are told that "the time seems ripe for a great forward movement against tuberculosis . . . and that here, as in other spheres, the future belongs to the brave." We trust that the editor will have such support as will enable him to carry on the campaign with the same energy and success as are shown by the January number, which should be read by all who take any interest in the attempt to combat tubercular diseases.

**The Sigmoidoscope.** By P. Lockhart Mummery, B.C. Cantab. Pp. 88. London: Baillière, Tindall & Cox. 1906.—This is an elementary treatise based upon the author's experience of his modification of Prof. Strauss's electric sigmoidoscope: the first half of which describes the methods of using the instrument, and the second half deals with some of the appearances met with. There appears to be a limited future for this method of diagnosis of diseases of the lower bowel which cannot be adequately discovered by other means, and the assistance of simultaneous air-inflation has diminished the dangers of sigmoidoscopy, and increased its possibilities. At the same time, surgeons of experience are well aware of the dangers attending the passage of long rigid instruments through the rectum by the inexperienced. The author records a case of perforation of the bowel into the peritoneum by the sigmoidoscope, although he says "the only danger which it seems to me might attend the use of the symoidoscope is that of tearing the mesentery of the sigmoid." It is doubtless an instrument of precision, essential in some cases to accurate diagnosis; but we predict that the average man will do wisely by leaving such examinations to those specially experienced, lest he be tempted to pass the sigmoidoscope as he might a bougie, with results disastrous to his patient and distressing to himself.

**Philadelphia Hospital Reports.** Vol. VI. Philadelphia: Bradley Printing Co. 1905.—The first paper is one of local interest, dealing with the first clinical reports issued by the Philadelphia Hospital. The other papers deal with various clinical subjects in reference to cases in the Hospital; and we are only doing justice to these papers when we say that they maintain a remarkably high standard, and will well repay perusal. The neurological articles perhaps especially deserve mention.

**Manual of Surgery.** By Alexis Thomson and Alexander Miles. Second Edition. Vol. I. Pp. xxi., 808. Edinburgh: Young J. Pentland. 1906.—The appearance of the second edition of this book in a little over two years testifies to its popularity. The first volume has been increased in size by forty odd pages. Some of the sections have been re-written, some of the old illustrations replaced by better ones, and some additional ones added. Specially is this noticeable in the orthopædic section at the end of the book. Also new are the photo-micrographs illustrating surgical bacteriology. These alterations and additions have added to the value of the work, which we still consider the best modern text-book on surgery for students.

**Glimpses of American Surgery in 1906.** By C. HAMILTON WHITEFORD. Pp. 63. London: Harrison and Sons. 1906.—This is quite an interesting booklet. In simple style we are told the impressions of the author at several of the American surgical clinics, notably of the Mayo Brothers, of Rochester, Minn.; of Murphy and Ochsner, of Chicago. The individuality of the various surgeons is nicely caught, and though we have heard a good many of the little trite sayings from the same mouths before, yet they will be new, interesting and often amusing to the profession in England.

**Spinal Curvatures.** By HEATHER BIGG. Pp. viii., 240. London: J. & A. Churchill. 1905.—This book has been written to advocate the mechanical as opposed to the gymnastic treatment of scoliosis. The author not only recommends a support for very advanced cases in which little good can be expected from exercises for the spine, and for which most surgeons would recommend one, but for all cases, at every stage of deformity. The general opinion of surgeons at the present time is that such mechanical supports have no curative action, but prevent further deformity, relieve pain, and should only be used for cases too severe for other methods of treatment. Heather Bigg, on the other hand, maintains that such mechanical support is curative, and he does not employ exercises to strengthen the spinal muscles, but applies an instrument to support the thorax on the pelvis, which is constantly worn in the day-time. We do not believe that any form of such an apparatus can mould the deformed spine straighter, but we do think that if the spine is supported in this way *during its period of growth* it may in time grow straight, or straighter, just as a bent tibia may when further bending is prevented by a suitable splint. We are therefore inclined to attach more importance to a supporting apparatus in the treatment of scoliosis than many surgeons, but we do not think that it should replace all other methods in cases which are not too advanced to be likely to derive benefit from them, and in which there is no marked change in the shape of the bodies of the vertebræ. Mr. Heather Bigg strongly objects to the gymnastic method as advocated by Mr. Bernard Roth and others, and he devotes a chapter to the consideration of Mr. Roth's treatment ; but we cannot say that he shows that the gymnastic treatment is valueless, though perhaps some surgeons have gone too far in denouncing the mechanical support. At any rate, it is interesting to read this original and well-written little book by Heather Bigg, and consider both sides of the question. The author goes very fully into the history of the treatment of scoliosis, and there are many good illustrations of cases and instruments in the book.

**Grundriss der Orthopädischen Chirurgic.** By Dr. MAX DAVID. Second Edition. Pp. v., 240. Berlin : S. Karger. 1906.—The first edition was published in 1900. The present one is considerably enlarged. It is essentially a practical work, most of the text being devoted to descriptions of the various deformities, and to carefully-described details of their treatment. The etiology and pathology are somewhat curtailed. The illustrations, although not of great artistic merit, serve well their purpose, and are very numerous. The authors quoted are for the most part German and Austrian, and comprise a very good selection. Compared with English surgeons, there is much more value attached to gradual reductions and forcible wrenchings of deformities, and also a greater use of plaster of Paris. The descriptions of the many complicated instruments with the aid of diagrams is clear and convincing ; these, especially in the cure of fixed joints, are much more used abroad than with us. The importance also of massage is not exaggerated. It is a work which would well repay translation, and be of considerable use to practitioners in England.

**The Sequelae of Gonorrhea in both sexes.** By W. LOUIS CHAPMAN, M.D. Pp. 117. Providence: Snow and Farnham. 1905. —A prize was awarded to the author for this essay by the Trustees of the Fiske Fund, Providence. As stated in the preface, it is largely a work of compilation of the most recently acquired knowledge on the subject plus investigation on the part of the author in some original directions. It deals very fully with the subject embraced. Commencing with the bacteriology, and showing that a gonotoxin may produce the symptoms apart from the actual organisms, a chapter is devoted to the mechanism of gonorrhœa and the importance of latent gonorrhœa. In dealing with the sequelæ common to both sexes, of special interest perhaps are the stomatitis of new-born infants, the various skin invasions, which may be of the nature of eczema, herpes, urticaria, &c., and the affections of the nervous system, neuritis, &c. In the sequelæ peculiar to the male, attention is paid to the part played by the prostate, and the possibility of gonorrhœal affection of that organ being a precursor to the enlargement of old age ; and in those peculiar to the female, peritonitis and the effect of gonorrhœa in producing sterility are the most noticable sections. Treatment is entirely omitted.

**Women's Health, and How to take care of it.** By FLORENCE STACPOOLE. Second Edition. Pp. viii., 165. Bristol: John Wright & Co. 1906.—This treatise is the outcome of much thoughtful experience in nursing the ailments peculiar to women, and is written in a plain, clear style that befits the subject. It may be warmly recommended to women who wish to know how to preserve their health, or to take care of the health of girls under their care. It will be specially useful to young women who have to live in lonely and distant parts of the world. The medical information is cleverly reproduced, but the distinction between anæmia and chlorosis is not clearly put. A particular excellence of the book is the appeal not to neglect the early symptoms—duly set forth—which may announce cancer of the uterus. We like the chapter on the climacteric period, and especially the advice as to diet ; but a few persons stint themselves too much to maintain an unnatural slimness. There is perhaps rather much prescribing, especially of iron, which should *never* be taken except under medical advice ; but probably nurses only will understand how to make use of the prescriptions. The book may be read with advantage by young medical practitioners.

**Medical Electricity.** By H. LEWIS JONES, M.A., M.D. Fifth Edition. Pp. xv., 519. London : H. K. Lewis. 1906.—Dr. Lewis Jones has added to the usefulness of his handbook by bringing it up to date. The author, himself a pioneer in the electrical world, gives what is best in the new work, and records new methods of usefulness for the old. The chapter on X-rays has been largely extended, and the electrical methods of dealing with various skin diseases also find a place. The experiments of Leduc in electrolysis are quoted, and a very useful addition to that subject is made in this present edition. Dr. Jones has been paying personal attention to the subject of introducing drugs into the body locally by electrolysis, and as much interest has been stimulated recently by the remarkable results obtainable in rodent ulcer by the introduction of the ions of zinc, it is valuable to have a treatise of reference for details of the technique. The handbook is so well supplied with illustrations, and the descriptions of apparatus, &c., are so easily followed, that we have no hesitation in recommending this book to any who wish to obtain some knowledge of the rapidly-extending subject of medical electricity.

**Climatotherapy and Balneotherapy.** By Sir HERMANN WEBER, M.D., and F. PARKES WEBER, M.D. Pp. 833. London: Smith, Elder & Co. 1907.—This volume on the climates and mineral water health resorts (spas) of Europe and North Africa, including the general principles of climatotherapy and balneotherapy, and hints as to the employment of various physical and dietetic methods, is practically a third edition of Sir H. Weber's book on the *Mineral Waters and Health Resorts of Europe*, but much enlarged in respect to medical climatology. Questions of climate and baths become of increasing importance as the facilities for rapid and easy travel annually increase, and books of this kind are soon out of date. Accordingly, we may welcome this volume as a very comprehensive summary of everything known on the subject, and giving the views of those whose experience must command universal regard. We cannot attempt a review of a book like this. It should be on the table for constant reference.

**The Röntgen Rays in the Diagnosis of Diseases of the Chest.** By HUGH WALSHAM, M.A., M.D., and G. HARRISON ORTON, M.A., M.D. Pp. 80. London: H. K. Lewis. 1906.—Dr. Walsham is well known as one of the pioneers in this country in applying the X-rays to the diagnosis of chest disease, and, in conjunction with Dr. Orton, he has given us this small volume, in which his experience is summarised. The use of the rays for diagnosing intra-thoracic disease is becoming much more general than it was, but the profession at large is still unaware to a great extent of its great value in diagnosis; and the authors have done well in drawing attention to this by saying that although it is now ten years since attention was drawn to the subject, "yet to-day the mass of practitioners in this country are quite ignorant of the value of the rays in the diagnosis of chest diseases." This little book ought to have a wide circulation, and will go far to remove this want of knowledge, which is much to be deplored. The value of the rays in diagnosing early phthisis is now generally admitted, and the chapter on this subject is one of the best in the book. The importance of the symptom of impaired movement of the diaphragm as seen on the fluorescent screen, which in many cases is present before any physical signs can be made out is rightly insisted on; and by skiagraphy it can often be demonstrated that disease has attacked both lungs, where only one shows signs of disease by physical signs. A number of cases illustrating these and other conditions are given. In the chapter on thoracic aneurism the difficulty and often the impossibility of diagnosis is pointed out; by means of skiagraphy, however, a correct diagnosis can practically always be made, the authors pointing out that to ensure a correct diagnosis the fluorescent screen must be used and the thorax skiagraphed in more than one position. This chapter, too, contains the records of interesting cases of aneurism thus diagnosed. This charmingly written little book is one of the most important contributions to the literature of skiagraphy that has appeared of late, and is cordially to be recommended to the notice both of X-ray workers—for the useful hints as to technique which it contains—and also to all members of the profession, as it points out to them the great assistance that they will receive from skiagraphy in all chest diseases, especially in those which are more or less obscure. It should be mentioned that the excellent reproductions of skiagrams, which elucidate the text, are quite one of the features of the book.

**Lessons on Massage.** By MARGARET D. PALMER. Third Edition. Pp. xvi., 272. London: Baillière, Tindall and Cox. 1907.—This book has had a large circulation, and several new chapters have been added in this edition. It is an instruction on the methods of massage; the elementary anatomy and physiology necessary for this is also included. As such it is clearly and concisely written, and easily understood by nurses, for whom it is especially meant. It does not pretend to advise as to which cases should have massage, or to discuss the reasons why the movements bring about the required results. Many conditions which are greatly benefited by this treatment are not even mentioned, and as it is no part of the trained masseuse to know them, it is probably wise that they have been omitted. One point insisted on is very important, namely the great advantage of working without lubricant or powder where possible. It would add to the utility of the work if some diagrams were introduced showing the direction and extent, by means of arrowed lines, of the various excursions made by the hands. Swedish movements are not dealt with, though these, and especially the respiratory exercises, ought to be known to every masseuse. The book just covers what is required in the examination of the Incorporated Society of Trained Masseuses.

**Lectures on Massage and Electricity in the Treatment of Disease.** By THOMAS STRETCH DOWSE, M.D. Sixth Edition. Pp. xii., 447. Bristol: J. Wright & Co. 1906.—We have been frequently called upon to review this book, and the sixth edition has not grown larger than the fourth. The author directs attention to the great and increasing appreciation of the value of massage as a remedial agent. His book has done much to bring about this result, but he regrets that the long-continued resistance to massage in this country has led to much abuse of this method in unqualified hands—"a good masseur should possess skill, intellect and judgment; but, above all, he must be a good manipulator." A close study of this book cannot fail to be of great value, both to those who prescribe and who perform massage and use electrical methods of treatment.

**The Uses of X-rays in General Practice.** By R. HIGHAM COOPER. Pp. x., 98. London: Baillière, Tindall and Cox. 1906.—**X-rays in General Practice.** By A. E. WALTER, Captain I.M.S. Pp. xii., 175. London: John Lane. 1906.—To quote the preface of the first of these books, "the intention of this little book is that of giving the general practitioner some idea of the help he may get in his practice from the use of the X-rays," and admirably it fulfils its object. It is quite a pleasure to come across a book so absolutely devoid of "padding," and to have a plain and straightforward account of the author's own method of work, and his experience of the use of the X-rays, both in diagnosis and treatment. After a masterly short summary of the physics of the X-ray, the author goes on to give in a most lucid way directions for working an induction coil with an improved pattern of the ordinary platinum break, wisely referring the reader to larger works on the subject for details of the more elaborate breaks which may be beyond the reach of the ordinary practitioner; and then proceeds to discuss the use of the rays in diagnosis and treatment. In the former most practical directions are given for taking skiagrams, such as the position of the tube as regards the part that is to be skiagraphed, length of exposure required, &c., &c., and in the latter the author confines himself entirely to a record of his own work, and gives the results of his own personal experience in the treatment of the various diseases in which the X-ray is used as a method of treatment. We can cordially recommend this little manual to all X-ray workers, not only to the novice, but also to the more advanced worker, who will find in its short 88 pages more that will be of practical use to them than in many volumes of much more pretentious size.

The second of these books is addressed to "the general practitioner, the student, and other non-experts in the X-rays." Like many other books on the subject now before the medical public, it gives advice as to choice of apparatus, how to set about taking a skiagram, &c.; but perhaps the most interesting part of the book is the account the author gives of the equipment he has devised for active service in the field. There are a number of most excellent illustrations; the one facing the title-page of a Chinese woman's foot, and of the skiagram of the same later on, are of special interest because of the difficulty in obtaining them, owing to its being "considered an act of the utmost indelicacy" for a Chinese woman to expose her foot. To the lover of personal detail, it may be of interest to have reproduced for his benefit the actual lesion which occurred when Lord Kitchener broke his leg—an ordinary fracture of the tibia and fibula. (We wonder whether Lord K. would be equally pleased?) But we do take exception to the statement on page 99: "His Excellency Lord Kitchener suffered a good deal when he first began to get about," and "he had a three weeks' course of high-frequency treatment, and I have his authority for saying that he was much benefitted thereby." This savours much more of the "Electropathic Institute" advertisement than of one of the volumes of the *Practitioner's Handbooks* Series.

**Transactions of the Epidemiological Society of London.** New Series. Vol. XXIV. Session 1904—1905. London: Williams and Norgate. 1905.—Amongst the interesting series of papers in this volume, probably the keenest interest will be felt in Prof.

MacWeeney's contribution, "On the Relation of the Parasitic Protozoa to each other and to Human Disease," and in Dr. Nuttall's "Ticks and Tick-transmitted Disease," which deal with a subject that is now undergoing very rapid growth as knowledge accumulates. Other subjects dealt with in this volume are "Ankylostomiasis," "The Etiology of Rheumatic Fever," "Phthisis Rates," "The Spread of Small-pox occasioned by Small-pox Hospitals," and the President (Dr. Whitelegge, C.B.) contributes a paper on "The Epidemiological Aspects of Industrial Disease."

**Reports of the Society for the Study of Disease in Children.** Vol. VI. Session 1905—1906. London: J. and A. Churchill.—The sixth volume of the reports of this young and vigorous Society fully maintains the high standard of excellence established by the preceding volumes. Dr. Whipham reports a case of splenic anæmia, which illustrates the difficulties of the anæmias of children when the splenic group is involved. Mr. Mackintosh contributes a common-sense paper on diet during the second year of life. We are surprised that a Society devoted to the study of disease in children does not pay more attention to the important question of feeding. The December meeting is occupied with a full-dress debate on "Pleural effusions, serous and purulent," which, owing to the eminence of the various speakers engaged, may be regarded as a succinct summary of our present knowledge of the subject. The Wightman Lecture was delivered by M. Broca, M.D., of Paris, who took for his subject "Appendicitis: acute and chronic." Dr. Bertram Rogers describes a case of acute atrophy of the liver. In a valuable paper on "Enlarged Veins in Children," Dr. A. G. Gibson points out the importance of enlarged veins on the thorax, in the diagnosis of enlarged tubercular mediastinal glands in children, when associated with other symptoms.

**Index-Catalogue of the Library of the Surgeon-General's Office, United States Army.** Second Series. Vol. XI.—Mo—Nyström. Pp. 858. Washington: Government Printing Office. 1906.—This volume includes 8,023 author-titles, 5,634 subject-titles of separate books and pamphlets, and 34,211 titles of articles in periodicals. It may help the uninitiated to form some idea of the immense amount of labour bestowed on the preparation of this invaluable work if we mention that under the headings of "Nerve," "Nerves," and "Nervous" there are about 155 pages of closely-printed references.

**Wellcome's Photographic Exposure Record and Diary, 1907.** London: Burroughs, Wellcome & Co. Pp. 268.—This popular pocket book has undergone its annual revision, and its information brought up to date. The simplicity and convenience of the mechanical calculator and light tables render the estimation of the correct exposure for any subject an easy matter. It is an excellent handbook for photographers generally.

**The Influence of Cod Liver Oil on Tuberculosis.** By J. W. Wells, M.D., D.P.H. Pp. 83. Manchester: The University Press. 1907.—Cod Liver Oil has for many years been looked upon as a most useful agent in the treatment of consumption. Of late it has been falling into disrepute, as it commonly forms no part of the programme of sanatorium treatment. The experiments recorded in this booklet, conducted in the Public Health Laboratory of the University of Manchester under the supervision of Professor Sheridan Delépine, tend to show that "pigs affected with tuberculosis continued to increase rapidly in weight, and appeared quite comfortable and happy for a long period when the Cod-liver Oil Emulsion was added to the usual diet. Their tubercle lesions showed signs of possible recovery, tuberculous glands became fibrous and calcified, and the tubercle bacilli more difficult to demonstrate." With this evidence before us, we may well ask whether the use of Cod Liver Oil in the treatment of phthisis should be discontinued.

**Guy's Hospital Reports.** Vol. LX. London: J. and A. Churchill. 1906.—The present volume opens with a valuable paper, by Dr. Frederick Taylor, on "The Chronic Relapsing Pyrexia of Hodgkin's Disease," in which he records nine cases where this disease was accompanied by relapsing pyrexia. He points out that the temperature may be continuously high for long periods, and that periods of higher fever may then alternate with periods of lower fever, and that the recognition of the relapsing form of pyrexia may be of assistance in the diagnosis of some doubtful cases. Dr. Herbert French and Mr. H. T. Hicks write on "Mitral Stenosis and Pregnancy," presenting in tabular form statistics of 300 consecutive cases of mitral stenosis in women over twenty who have been in Guy's Hospital. They consider that the dangers of pregnancy in these cases have been overstated, and that it is not just to negative marriage in all women with mitral stenosis. Two highly interesting lectures, delivered at the Physiological Laboratory, Guy's Hospital, by Dr. J. S. Haldane—a brother of the present War Minister—on "Life and Mechanism," are here published. Dr. Haldane considers that while the old vitalistic working hypothesis in physiology was altogether unsatisfactory, the mechanistic hypothesis which some fifty years ago replaced it, is inconsistent with observed phenomena, and must also be rejected. In biology we cannot get beyond the fundamental working conception of the living organism, which is an organism, and not a machine. The lectures are expressed in admirable language, and are worthy of thoughtful study. We have no space to notice the other articles in this volume, which are of unusual importance and interest.

